# Hypoxia-inducible factor 1 alpha promotes cancer stem cells-like properties in human ovarian cancer cells by upregulating SIRT1 expression

**DOI:** 10.1038/s41598-017-09244-8

**Published:** 2017-09-06

**Authors:** Jie Qin, Yan Liu, Yongkui Lu, Meiling Liu, Manli Li, Juan Li, Lijuan Wu

**Affiliations:** 1 Obstetrics and gynecology, Center for reproductive medicine and genetics. The people’s hospital of Guangxi Zhuang Autonomous Region, Nanning, People’s Republic of China; 2 The Department of breast and bone tissue oncology, Affiliated Tumor Hospital of Guangxi Medical University, Nanning, People’s Republic of China

## Abstract

Ovarian cancer have a poor overall survival rate in patients, and late disease presentation and chemoresistance are the main factors that lead to the mortality of ovarian cancer. Cancer stem cells (CSCs), a small subpopulation of cancer cells, have been associated with resistance to chemo- and radio-therapy in cancer treatment. Hypoxia is a common characteristic of many malignant tumors, and increased HIF-1α expression predicts the poor prognosis of ovarian cancer. In this study, we reported the relationship between hypoxia and cancer stem cells-like properties in human ovarian cancer cell lines SKOV3 and HO8910, we found that hypoxia induced cancer stem cells-like properties in ovarian cancer cells. Moreover, SIRT1 was found to be the downstream target gene of HIF-1α, which was involved in the promotion of cancer stem cells-like features in ovarian cancer cells by hypoxia, and NF-κB signaling pathway was involved in hypoxia-induced SIRT1 up-regulation. Our results hinted that HIF1α and SIRT1 might serve as potential therapeutic targets for ovarian cancer.

## Introduction

Ovarian cancer has been described as the seventh most common female cancer worldwide^1^. According to the research in 2013, the 5-year overall survival rate of patients with ovarian cancer is less than 40%^2^. Although early-stage ovarian cancer can be successfully removed with surgery alone, most patients are diagnosed at the advanced stages. The mortality rate for ovarian cancer is quite high because of late disease presentation and chemoresistance^1^. While the majority of patients initially respond well to chemotherapy, many patients relapse and become chemoresistant, which leads to insurmountable obstacles for improving the survival rate^3, 4^.

Cancer stem cells (CSCs), a small subpopulation of cancer cells, which possess self-renewal and multilineage differentiation potential^5^. A lot of malignant tumors contain these highly tumorigenic and intrinsically drug-resistant CSCs. Now several molecules such as CD44, CD133, CD24, Oct4 and Nanog have been reported to be related with CSCs, and high level of these molecules was associated with resistance to chemo- and radio-therapy in cancer treatment^5–7^. Therefore, therapeutic strategies that specifically target CSCs are likely to be effective in ovarian cancer and in reducing the chemoresistance of ovarian cancer.

Hypoxia is a common characteristic of many cancers^8^. Hypoxia-inducible factor 1 (HIF-1) is a transcription factor that is induced under hypoxia condition^9^. And downstream target genes and pathways of HIF-1 will be activated to regulate the cell response due to a hypoxia treatment^10^. HIF-1 is a heterodimeric protein consisting of HIF-1α and HIF-1β. HIF-1α expression is usually maintained at low levels under normoxic conditions and which will be significantly induced by hypoxia^11, 12^. HIF1-α has been demonstrated to be an key predictor of tumor progression for several types of solid cancers^13^. And increased HIF-1α expression has also been linked to poor survival in clinical^14^. Additionally, hypoxia also endow tumor cells with a stem cell-like properties and promote cells to survive in the poor tumor microenvironment^15^. However, the mechanism is still unclear.

We reported here that hypoxia induced CSCs-like characteristics in human ovarian cancer cell lines SKOV3 and HO8910. Moreover, Sirtuin type 1 (SIRT1) was found to be the downstream target gene of HIF-1α, which was involved in the promotion of CSCs-like features in ovarian cancer cells induced by hypoxia. The results of this study indicated that in ovarian cancer HIF1α and SIRT1 might serve as potential therapeutic targets.

## Material and Method

### Ethics statement

The procedure of animal experiment was approved by the Animal Care and Experimentation Committee of Guangxi Medical University. All methods were carried out in accordance with the approved guidelines.

### Culture of human ovarian cell lines

SKOV3 and HO8910 cell lines were purchased from the Shanghai Cell Bank of Chinese Academy of Sciences. The cells were cultured in McCoy’s 5A medium (Sigma, St. Louis, MO, USA) supplemented with 10% fetal bovine serum (FBS, Gibco, Australia), 100 lg/ml streptomycin and 100 units/ml penicillin. Cultures were maintained at 37 °C in a humidified atmosphere of 5% CO_2_. The oxygen concentration of hypoxia condition was 1% O_2._

### Antibodies and siRNA transfection reagents

The monoclonal antibodies used for Vimentin and E-cadherin immunofluorescence staining were purchased from BD Biosciences (Mississauga, ON). The antibody for HIF-1α detection was obtained from Novus Biologicals (Littleton, CO). The antibodies used for the examination of CD133, CD44, Nanog, GAPDH, SIRT1, IκBа and p-IκBа were obtained from Cell Signaling Technology (Danvers, MA). The second antibodies including goat anti-mouse IgG antibody and goat anti-rabbit IgG antibody were purchased from Bioworld Technology (MN 55416, USA). The siRNA reagents that used for gene inhibition including transfection medium, control sequences, HIF-1α and SIRT1 were obtained from Santa Cruz Biotechnology (Carlsbad, CA, USA). BAY11-7082 which was NF-κB specific chemical inhibitor was from Selleck Chemicals (USA).

### Real-time PCR (RT-PCR)

According to the protocol from manufacture, trizol (Invitrogen, Carlsbad, CA, USA) was used to isolate total mRNA and PrimeScript RT reagent Kit (Takara, Kyoto, Japan) was used to synthesis complementary DNA. Then realtime-PCR was performed by using SYBR Green PCR Kit (Applied BI). The reaction included 95 °C for 10 minutes, which was followed by a two-step PCR program of 95 °C for 15 seconds and 60 °C for 30 seconds repeated for 40 cycles.

### Western blots

Total protein was extracted from cultured cells and concentrations of protein were examined by using the DC Protein Assay kit with BSA as the standard (Bio-Rad Laboratories). The protein were separated equally by SDS polyacrylamide gel electrophoresis and transferred to PVDF membranes. Tris-buffered saline containing 5% non-fat dry milk was employed to block the unspecific binding site of the protein for 1 hour. After that the membranes were incubated with primary antibodies overnight at 4 °C and then followed by incubation with secondary antibody conjugated by HRP. An enhanced chemiluminescent substrate (Pierce, Rockford, IL) was used to observe immunoreactive bands. At last, the membranes were washed with stripping buffer for 30 min at 50 °C and GAPDH was employed as an internal control to confirmed the amount of protein.

### Cell counting kit-8 assay

The cell viability was examined by cell counting kit-8 (CCK-8) (Dojindo Laboratories, Kumamoto, Japan)^16^. Firstly, the cells (5 × 10^3^) were plated into 96-well plates each well and cultured overnight. Then the cells were exposed to chemoreagents including cisplatin (10 μg/ml) and 5-fluorouracil (5-Fu, 150 μg/ml) for 48 h, respectively. After chemotherapy, 200 μl medium containing CCK-8 and serum-free medium (v/v 1/10) were added in each well. Then the cells were incubated in a humidified atmosphere containing 5% CO_2_ at 37 °C. At last, the OD value of each well was examined by using a microplate reader at 450 nm (Zenyth 3100, Anthos, UK).

### Transwell assay

In order to perform a transwell assay, matrigel invasion chambers coated with 8.0 μm PET membrane in 24 well plates (Corning, USA). Matrigel invasion chambers were pre-incubated with 50 µl medium for 30 minutes at 37 °C. Then the cells (5 × 10^4^) in the 150 µl medium were plated in upper chamber. Meanwhile 500 µl medium containing 5% FBS was added in the lower chamber. After 36 hours of incubation, the cells on the upper surface were wiped with cotton swab. The cells on the lower surface of the filter were fixed with 4% formaldehyde for 15 minutes and then stained with 0.1% crystal violet dye for 30 minutes. Stained cells were observed and counted by microscope.

### Animal model

Female athymic BALB/c nu/nu mice (six-week-old) were purchased from Shanghai Experimental Animal Center, Chinese Academy of Science. Mice were maintained in a pathogen-free condition. 5 × 10^4^ cells were injected subcutaneously into the right armpit area of mice. The mice were sacrificed for analysis after 4 weeks. Tumor volume was calculated (ab^2^/2), where a was the length of the tumor and b was the tumor width^17^. All animal experiments were approved by the Animal Care and Experimentation Committee of Guangxi Medical University.

### Cell immunofluorescence

Cells were plated in 24-well plate overnight. Then the cells in each well were fixed with 4% paraformaldehyde for 15 min, followed by exposure with 0.1% Triton for 10 min. Then cells were disposed with PBS (containing 1% FBS) at 37 °C for 30 min and target protein specific primary antibodies were added in wells and incubated overnight at 4 °C. Then the cells were washed with PBS, after that, samples were treated with conjugated secondary antibodies at 37 °C for 30 min in the dark. Nuclei were stained with DAPI for 2 min. After washing, the cells were keep in PBS and observed by Olympus ZX71 microscope (Olympus Corp., Japan).

### Small interfering RNA (siRNA) transfection

Small interfering RNA (siRNA) was used to knockdown endogenous HIF-1α and SIRT1 according to the manufacturer’s instructions. 2 × 10^5^ ovarian cancer cells were seeded in each well of six-well plate. siRNA transfection reagent mixture (solution A + solution B) were added in each well for the transfection. Then HIF-1α and SIRT1 siRNA transfection medium (0.8 ml) was added to each tube and then incubated for an additional 24 hours.

### Statistical analysis

Data were analyzed by GraphPad Prism 5.0 (GraphPad Software). Results are presented as mean ± SEM of at least three independent experiments. Quantitative data were showed as mean ± SD in each performance. Significance between groups was performed using Student’s t test. P < 0.05 was considered as statistical significances.

## Results

### Hypoxia treatment promoted the expression of CSCs’ markers in ovarian cancer cells

**Figure 1:**
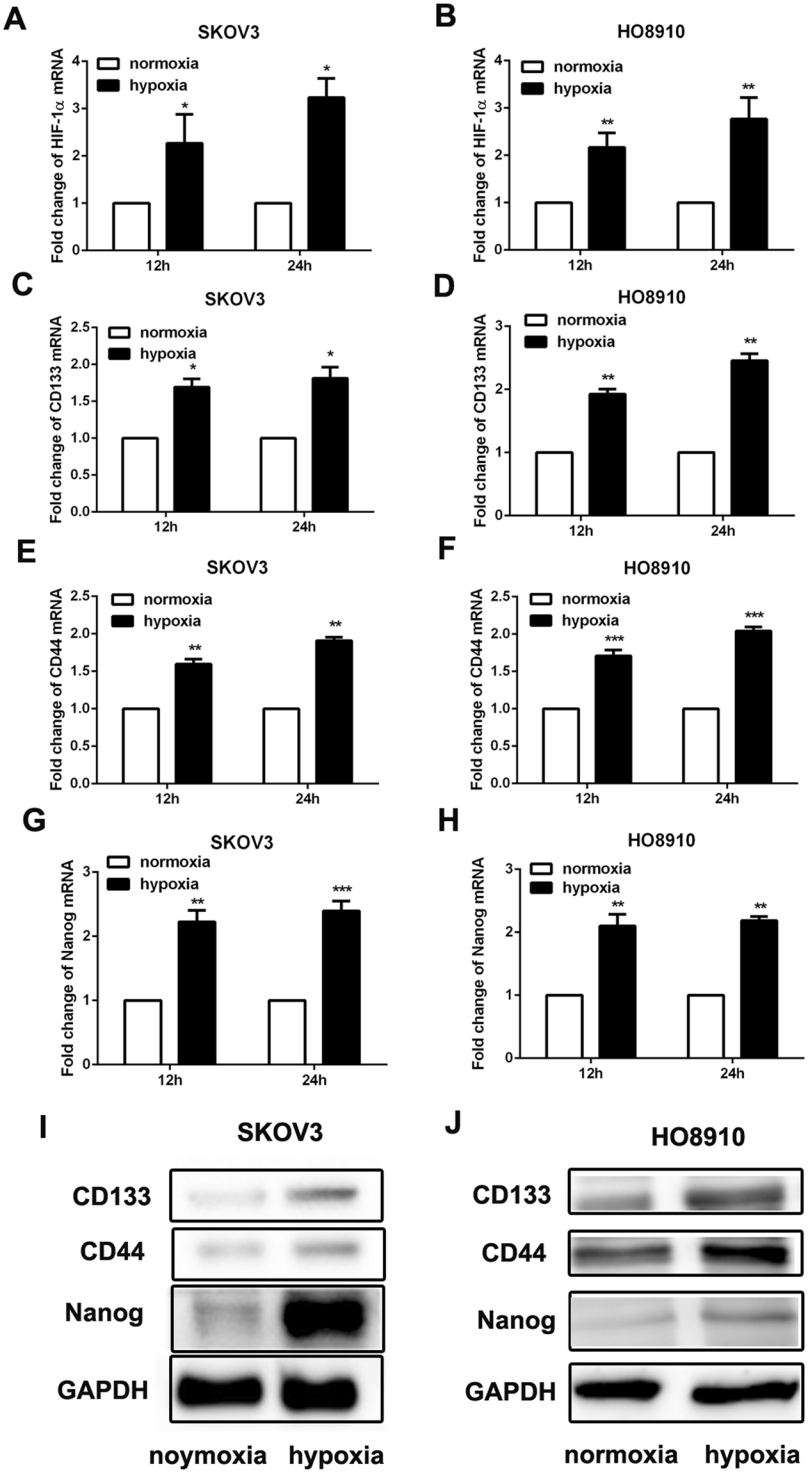
Hypoxia treatment promoted the expression of CSCs’ markers in ovarian cancer cells. (**A**–**H**) RT-PCR was performed to detect the expression of CD133, CD44 and Nanog in ovarian cancer cells SKOV3 and HO8910 after 12 h and 24 h of hypoxia treatment, GAPDH was used as an internal reference. *p < 0.05, **p < 0.01, ***p < 0.001. (**I** and **J**) Western blot was used to examine the expression of CD133, CD44 and Nanog in ovarian cancer cells SKOV3 and HO8910 after 24 h of hypoxia treatment. GAPDH was used as an internal reference.

Ovarian cancer cell lines SKOV3 and HO8910 were incubated at 1% O_2_. HIF-1α was tightly regulated in hypoxic conditions (1% O_2_) (Fig. 1A and B). Then the expression of CD133, CD44 and Nanog was detected by RT-PCR and western blot. And we found that CD133, CD44 and Nanog mRNA expressed at a significant higher level in ovarian cancer cell lines SKOV3 and HO8910 exposed for 12 and 24 hours to 1% O_2_ than that in normoxia (20% O_2_) (Fig. 1C–H). And the same results were also examined from western blot (Fig. 1I and J). These data showed that hypoxia condition could promote the CSCs’ markers expression in ovarian cancer cells.

### Hypoxia treatment induced the chemoresistance and tumorigenicity in ovarian cancer cells

**Figure 2:**
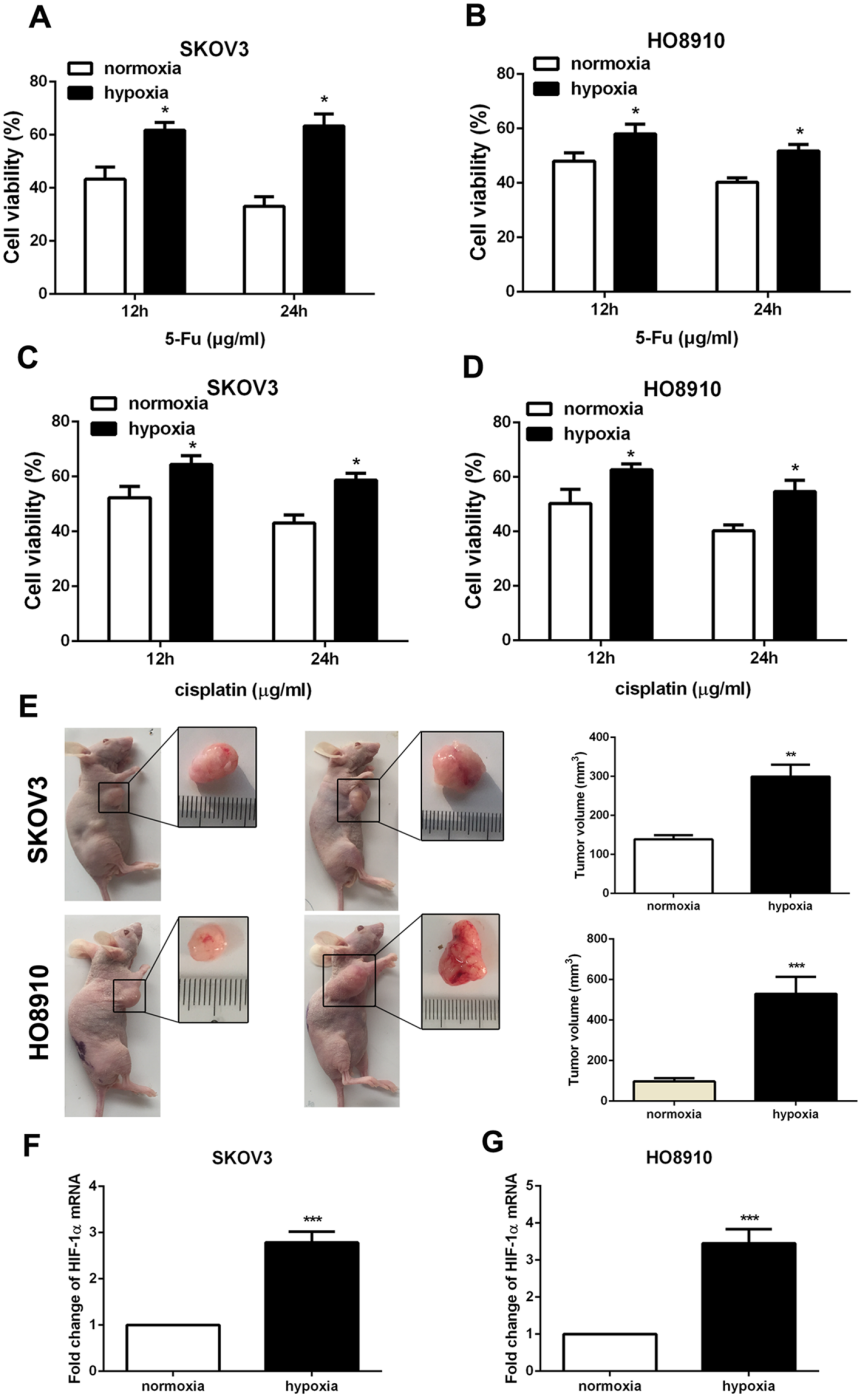
Hypoxia treatment induced the chemoresistance and tumorigenesis in ovarian cancer cells. (**A**–**D**) Cell viability of SKOV3 and HO8910 cells with chemotherapy under normoxia and hypoxia condition was determined by CCK-8 assay. Data of three replicates are shown as means (±SD). *P < 0.05. 5-Fu: 5-fluorouracil. (**E**) Tumorigenesis of SKOV3 and HO8910 cells was detected after cultured with hypoxia for 24 h. Pictures were taken 4 weeks after subcutaneous injection. Quantitative analysis of tumor volume in each group is listed. Data of three replicates are shown as means (±SD). ***P < 0.001. (**F** and **G**) The expression of HIF-1α was detected by RT-PCR in tumor. Data of three replicates are shown as means (±SD). ***P < 0.001.

Chemoresistance is the main feature of ovarian cancer. In order to examine the influence of hypoxia on the capacity of chemoresistance in ovarian cancer cells, 5-Fu and cisplatin were used to treat ovarian cancer cells. The data of CCK-8 experiment showed that cells with hypoxia-treated have higher cell viability compared to those in control at 12 and 24 h of culture with 5-Fu or cisplatin (Fig. 2A–D). These results hinted that hypoxia have a key role in the enhancement of chemoresistance in ovarian cancer cells. Moreover, ovarian cancer cell lines SKOV3 and HO8910 were subcutaneously injected in BABL/c nude mice. And the *in vivo* tumor development experiment showed that hypoxia-disposed ovarian cancer cells displayed significantly stronger ability of tumorigenesis than those without hypoxia stimulation (Fig. 2E). Additionally, HIF-1α expression was at a high level in tumor from hypoxia-pretreatment ovarian cancer cells (Fig. 2F and G).

### Hypoxia treatment induced invasion of ovarian cancer cells

**Figure 3:**
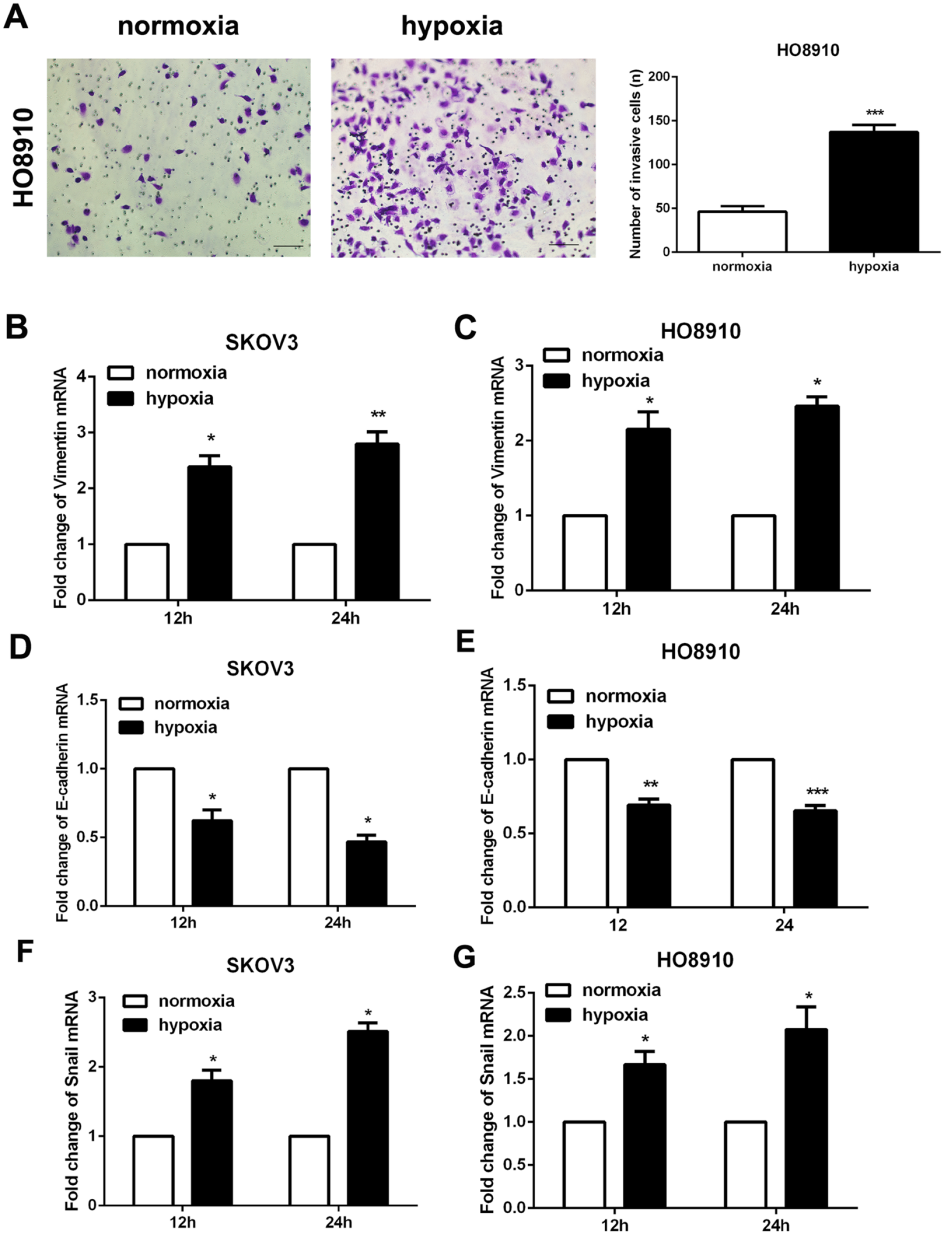
Hypoxia treatment induced epithelial to mesenchymal transition (EMT) phenotype in ovarian cancer cells. (**A**) Transwell assay was used to detect the invasion of ovarian cancer cell line HO8910. Data of three replicates are shown as means (±SD). ***P < 0.001. (**B**–**G**) RT-PCR was performed to detect the expression of Vimentin, E-cadherin and Snail in ovarian cancer cells SKOV3 and HO8910 after 12 h and 24 h of hypoxia treatment, GAPDH was used as an internal reference. *p < 0.05, **p < 0.01, ***p < 0.001.

**Figure 4:**
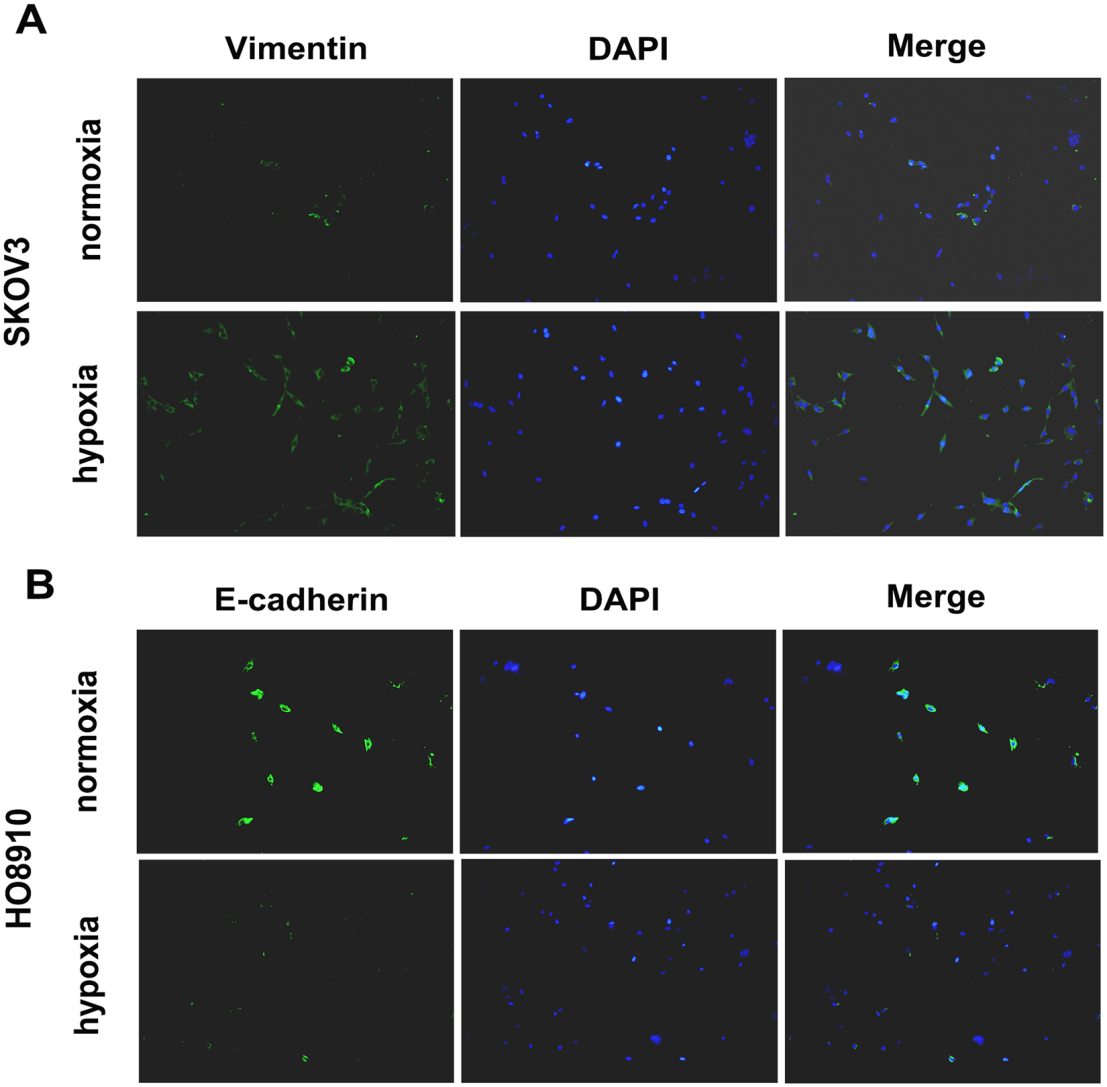
Hypoxia induced epithelial to mesenchymal transition (EMT) phenotype in ovarian cancer cells. (**A** and **B**) Immunofluoresence was used to evaluate the expression of Vimentin and E-cadherin in SKOV3 and HO8910 cells.

Next, transwell assay was performed to detect the invasion of ovarian cancer cells. The results demonstrated that hypoxia cloud significantly promote the invasive ability of HO8910 cells (Fig. 3A). EMT is a process that switches from epithelial phenotype to mesenchymal phenotype, which enable these cells acquire migratory and invasive features^18^. Several research has demonstrated that EMT was a main feature of CSCs^19^. Therefore, EMT phenotype was detected in ovarian cancer cell lines SKOV3 and HO8910 cultured in hypoxia condition after 12 h and 24 h. The RT-PCR results showed that the mesenchymal marker (Vimentin) and transcription factor (Snail) were upregulated and epithelial marker (E-cadherin) was downregulated in hypoxia-treatment ovarian cancer cells (Fig. 3B–G). And cell immunofluorescence results also showed that Vimentin expression was increased and E-cadherin expression was decreased in hypoxia-treatment SKOV3 and HO8910 cells (Fig. 4A and B).

### CSCs-like properties were decreased by interfering HIF1α expression in hypoxia condition

**Figure 5:**
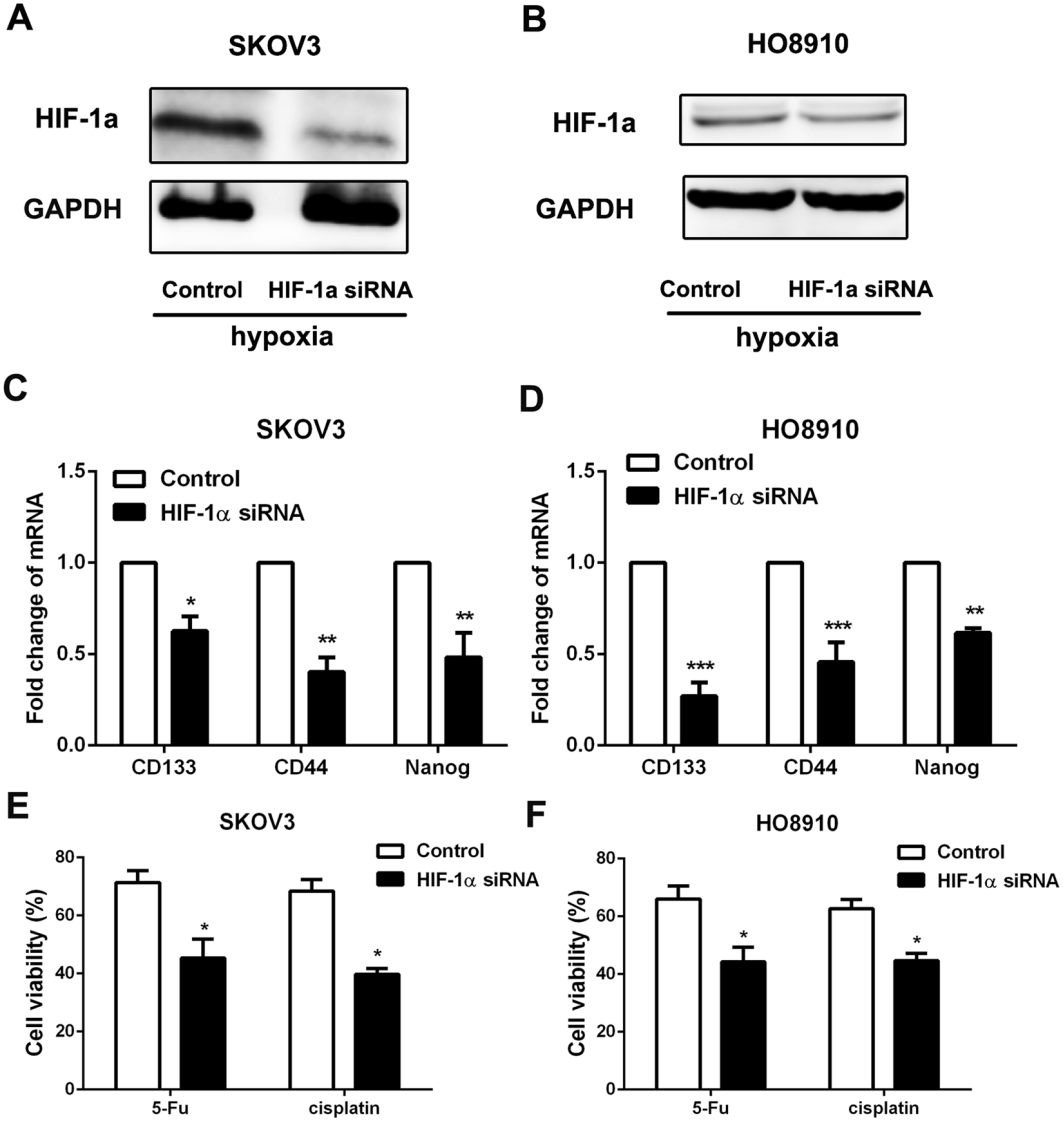
The expression of CSCs’ markers and chemoresistance were decreased by interfering HIF1α expression in hypoxia condition. (**A** and **B**) The expression of HIF-1α was confirmed after using HIF-1α-specific siRNA by western blot, GAPDH was used as an internal reference. Control siRNA was used as control. (**C** and **D**) RT-PCR was performed to detect the expression of CD133, CD44 and Nanog in ovarian cancer cells SKOV3 and HO8910 after 24 h of HIF-1α siRNA treatment in hypoxia condition. GAPDH was used as an internal reference. Control siRNA was used as control. *p < 0.05, **p < 0.01, ***p < 0.001. (**E** and **F**) Cell viability of SKOV3 and HO8910 cells with chemotherapy after 24 h of HIF-1α siRNA treatment in hypoxia condition was determined by CCK-8 assay. Data of three replicates are shown as means (±SD). *P < 0.05. 5-Fu: 5-fluorouracil.

**Figure 6:**
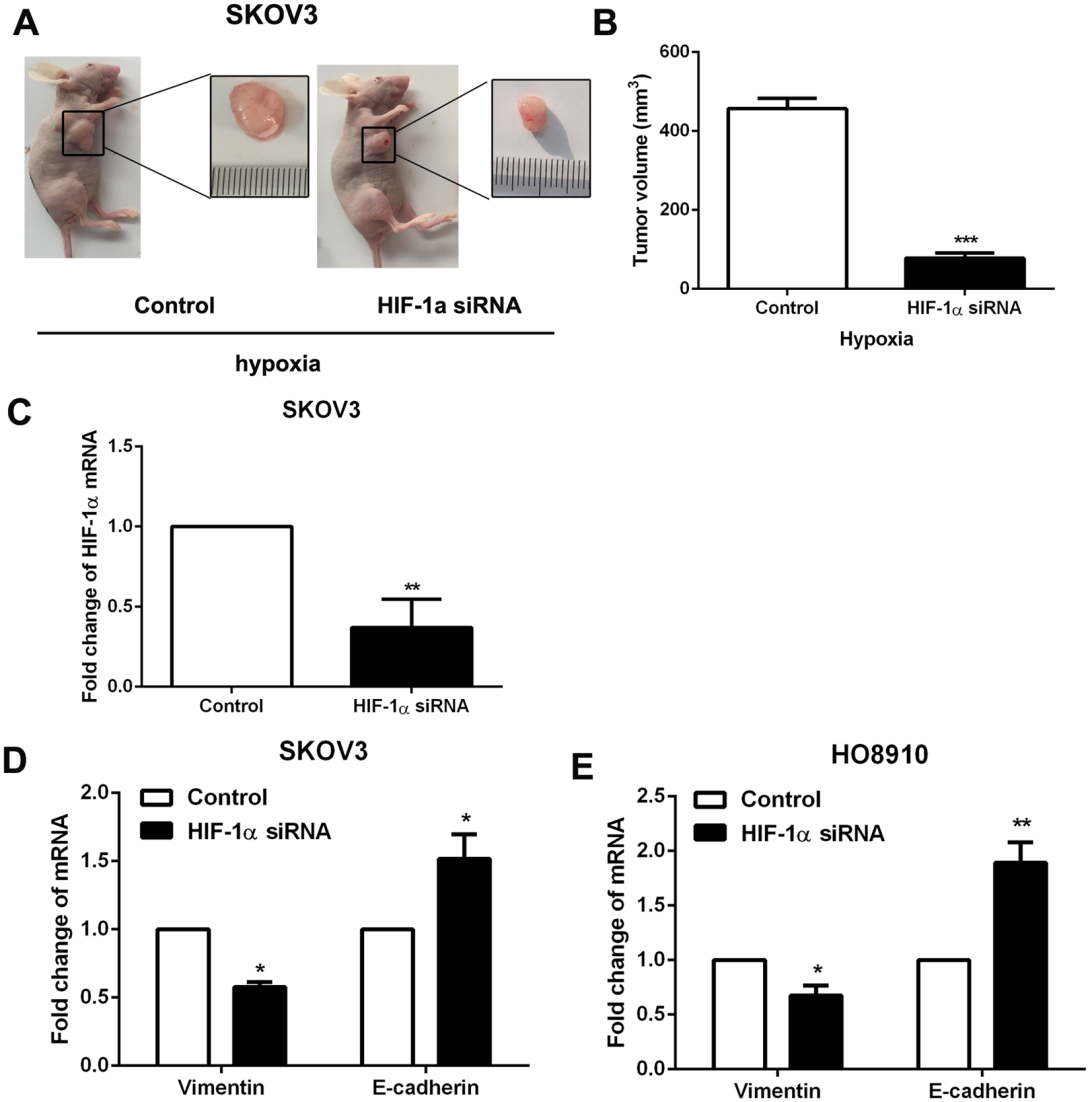
The tumorigenicity and EMT phenotype were decreased by interfering HIF1α expression in hypoxia condition. (**A**) Tumorigenicity of SKOV3 cells was detected after 24 h HIF-1α siRNA treatment in hypoxia condition. Pictures were taken 4 weeks after subcutaneous injection. (**B**) Quantitative analysis of tumor volume in each group is listed. Data of three replicates are shown as means (±SD). ***P < 0.001. (**C**)The expression of HIF-1α was detected by RT-PCR in tumor. Data of three replicates are shown as means (±SD). ***P < 0.001. (**D** and **E**) RT-PCR was performed to detect the expression of Vimentin and E-cadherin in ovarian cancer cells SKOV3 and HO8910 after 24 h of HIF-1α siRNA treatment in hypoxia condition. GAPDH was used as an internal reference. Control siRNA was used as control. *p < 0.05, **p < 0.01, ***p < 0.001.

To determine the relationship between HIF-1α and CSCs-like features, HIF-1α-specific siRNA (0.75 μg/ml) was used to decrease HIF-1α expression in ovarian cancer cells under hypoxia condition (1% O_2_). And then the expression of CSCs’ markers, chemoresistance, tumorigenesis and EMT phenotype were detected. The specificity of siRNA was examined with western blot, and the results confirmed that the siRNA used was specific and could reduce the HIF-1α expression in SKOV3 and HO8910 cells (Fig. 5A and B). The data showed that knockdown of HIF-1α expression cloud significantly reduced the expression of CSCs’ markers (Fig. 5C and D), chemoresistance (Fig. 5E and F), tumorigenesis (Fig. 6A–C) and EMT phenotype (Fig. 6D and E) in ovarian cancer cells. The results above demonstrated that there was a significant correlation between HIF-1α expression and CSCs-like features in ovarian cancer.

### HIF-1α promoted the CSCs-like features by increasing SIRT1 expression via NF-κB signaling pathway activation

**Figure 7:**
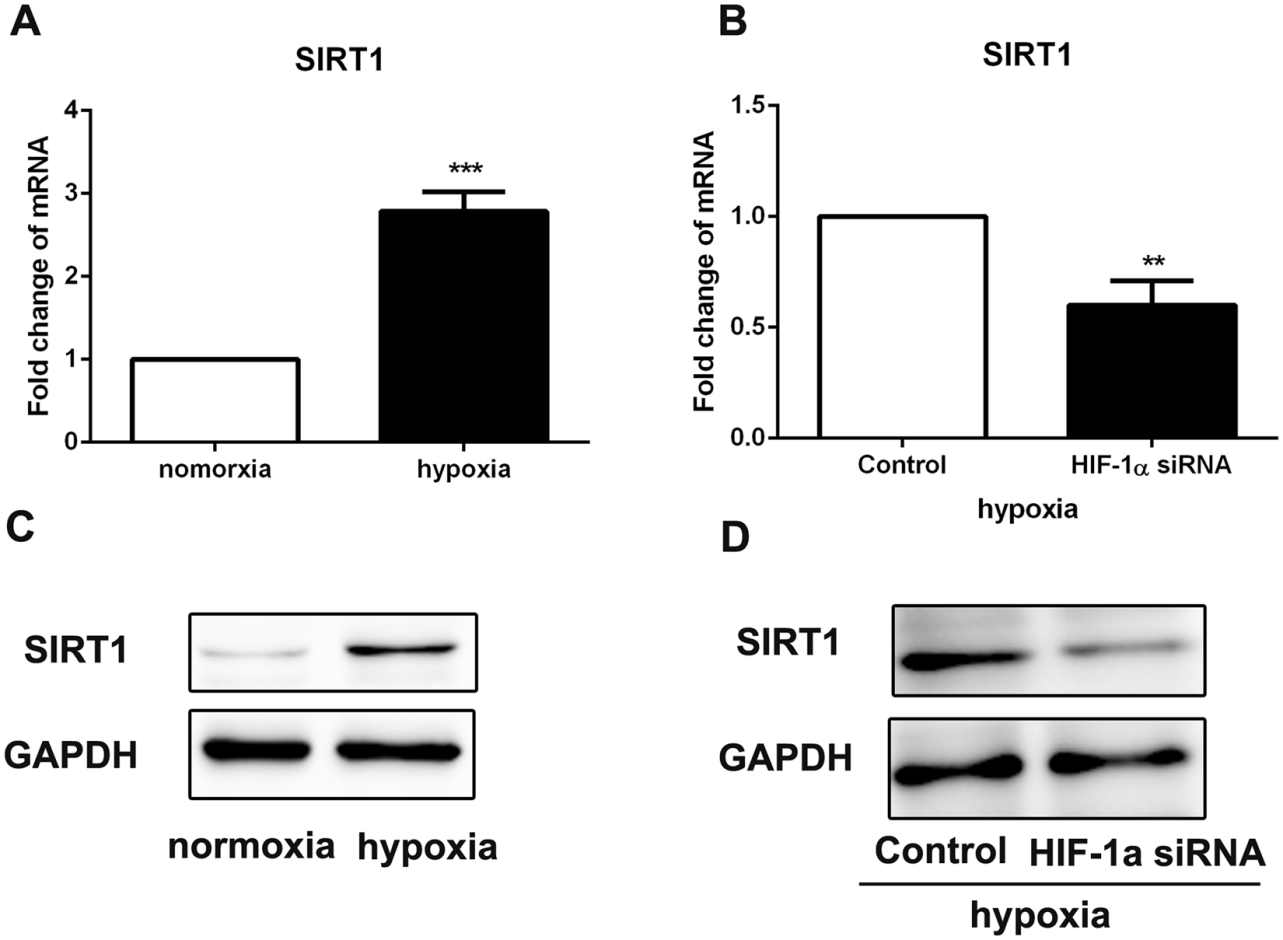
HIF-1α unpreguated SIRT1 expression in ovarian cancer cells. (**A** and **B**) RT-PCR was performed to detect the expression of SIRT1 in ovarian cancer cells SKOV3 after 24 h of hypoxia treatment or HIF-1α siRNA treatment in hypoxia condition. GAPDH was used as an internal reference. Control siRNA was used as control. **p < 0.01, ***p < 0.001. (**C** and **D**) Western blot was used to detect the expression of SIRT1 in ovarian cancer cells SKOV3 after 24 h of hypoxia treatment or HIF-1α siRNA treatment in hypoxia condition. GAPDH was used as an internal reference. Control siRNA was used as control.

**Figure 8:**
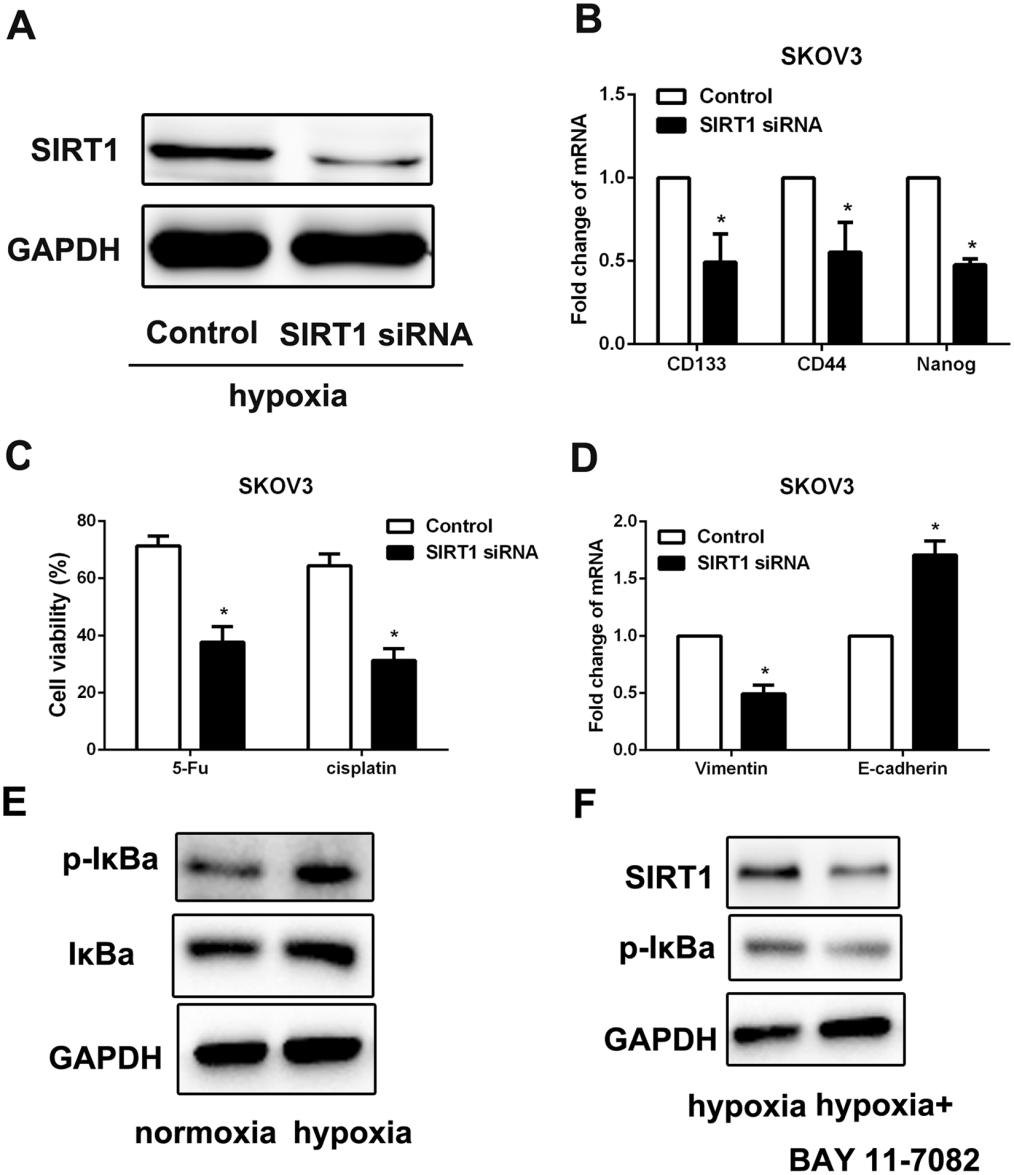
HIF-1α promoted the CSCs-like features by increasing SIRT1 expression via NF-κB signaling pathway activation. (**A**) The expression of SIRT1 in SKOV3 cells was confirmed after using SIRT1-specific siRNA by western blot, GAPDH was used as an internal reference. GAPDH was used as an internal reference. Control siRNA was used as control. (**B**) RT-PCR was performed to detect the expression of CD133, CD44 and Nanog in SKOV3 cells after 24 h of SIRT1 siRNA treatment in hypoxia condition. GAPDH was used as an internal reference. Control siRNA was used as control. *p < 0.05. (**C**) Cell viability of SKOV3 cells with chemotherapy after 24 h of SIRT1 siRNA treatment in hypoxia condition was determined by CCK-8 assay. Data of three replicates are shown as means (±SD). *P < 0.05. 5-Fu: 5-fluorouracil. (**D**) RT-PCR was performed to detect the expression of Vimentin and E-cadherin in ovarian cancer cells SKOV3 cells after 24 h of SIRT1 siRNA treatment in hypoxia condition. GAPDH was used as an internal reference. Control siRNA was used as control. *p < 0.05. (**E**) Western blot was used to detect the activation of NF-κB signaling pathway. GAPDH was used as an internal reference. (**F**) Western blot was used to detect the expression of SIRT1 when NF-κB signaling pathway was blocked by the inhibitor BAY11-7082. GAPDH was used as an internal reference.

SIRT1 has been reported as a downstream target gene^20^ and suggested to have a critical role in tumorigenesis^21^, Therefore, we examined SIRT1 expression in ovarian cancer cells cultured in hypoxia condition and these transfected with HIF-1α siRNA. As expected, SIRT1 expression was unregulated by hypoxia treatment and was downregulated by HIF1α siRNA disposure  in SKOV3 cells by RT-PCR and western blot examination (Fig. 7A–D). To determine whether SIRT1 was involved in the promotion of CSCs-like characteristics induced by HIF-1α, we transfected SIRT1 siRNA in SKOV3 cells and observed a dramatic decrease in SIRT1 protein levels in hypoxia condition (Fig. 8A). We found that expression of CSCs’ markers was downregulated by SIRT1 siRNA in SKOV3 cells (Fig. 8B). Moreover, SKOV3 cells transfected with SIRT1 siRNA exhibited a significant decrease in chemoresistance and EMT phenotype (Fig. 8C and D). These results indicated that SIRT1 was involved in the increase of CSCs-like features in ovarian cancer cells induced by HIF-1α.

Next, the mechanism between HIF-1α and SIRT1 was detected. We found that NF-κB signaling pathway was activated by hypoxia (Fig. 8E). In order to detect the activation of NF-κB was involved in SIRT1 upregulation which induced by hypoxia. BAY11-7082 (a NF-κB inhibitor, 10 μM) was used to block NF-κB pathway. And the results showed that blocking NF-κB pathway cloud significantly inhibit SIRT1 expression in hypoxia-stimulated cells (Fig. 8F). These results indicated that the hypoxia induced CSCs-like properties was supported by SIRT1 up-regulation through activation of NF-κB pathway.

## Discussion

Patients with ovarian cancer initially respond well to surgical treatment and the recurrence and poor survival are mainly due to the chemoresistance, usually drug-resistant disease that is because of ovarian cancer stem cells. Cancer stem cells contribute to the chemoresistance and metastasis of ovarian cancer, and give rise to tumor formation. Thus, development of therapeutic strategies that can target CSCs is benefit for improving the survival of patients, especially those with clinical chemotherapy and metastasis. Here we provided the evidence that HIF-1α and its downstream target gene SIRT1 played an important role in the promotion of CSCs-like properties in ovarian cancer cells.

Recent study have identified that HIF-1α played an important role in the process of tumor adaptation to hypoxia^22^, and HIF-1α expression was associated with tumor invasion, metastasis, poor prognosis and resistance to treatment^23^. In ovarian cancer, HIF-1α expression is more frequent in malignant than in benign ovarian tumors^14, 24^. And strongly staining for HIF-1α has been found in ovarian cancer patients with poor survival^24^. These studies suggest that the prognosis of patients with serious ovarian cancer may be evaluated by examining the expression of HIF-1α. Our present studies have found that the high expression of HIF-1α promoted CSCs-like features in ovarian cancer cells, including CSCs’ markers expression, chemoresistance, tumorigenesis and EMT phenotype. Our data indicated that HIF-1α contributed to the malignance of ovarian cancer for promoting CSC-like characteristics.

SIRT1, a member of the sirtuin family, is a kind of nicotinamide adenine dinucleotide (NAD+)-dependent histone deacetylase^25^. High expression of SIRT1 has been found in several cancers including, breast cancer, prostate cancer, lung cancer, colon cancer, and gastric cancer, which specific function is involved in cell proliferation, survival, differentiation and carcinogenesis^26–30^. It has been reported that SIRT1 modulates the activation of HIF-1α^31^. However, SIRT1 was also found to be strongly increased in cells exposed to repetitive cycles of hypoxia^30, 32^. In our work, we found that SIRT1 was overexpressed in ovarian cancer cells exposure to hypoxia condition, and its expression was regulated by HIF-1α. Additionally, we found that NF-κB signaling pathway was involved in the upregualtion of SIRT1 which induced by HIF-1α. Therefore, It is not only that SIRT1 could regulate HIF-1α expression, HIF-1α could also mediate the activation of SIRT1. Additionally, over-expression of SIRT1 has been found to induce chemoresistance and poor prognosis of ovarian cancer^33^. And there are several reports show that SIRT1 could induce EMT in several tumors^34, 35^. To further determine whether SIRT1 is involved in the CSC-like properties induced by HIF-1α, we silenced the expression of SIRT1 by siRNA. And our data indicated that SIRT1 was involved in the high expression of CSCs’ markers, chemoresistance, tumorigenesis and EMT phenotype. In this context, the inhibition of SIRT1 is becoming a novel approach for new treatment strategies of ovarian cancer. Additionally, our data suggested that an additional benefit of targeting HIF-1α would be the inhibition of SIRT1 activity.

Our research demonstrated that high level of HIF-1α and SIRT1 was related to the characteristics of CSCs in ovarian cancer. Our data suggested that HIF-1α and SIRT1 could be predictors for chemoresistance and prognosis of ovarian cancer and they will be the targets for the development of new therapies for ovarian cancer. Moreover, we found that SIRT1 was a main target of HIF-1α through activating NF-κB pathway. Understanding the connection between HIF-1α and SIRT1 is complex and the current literature is in part, and it still needs further evidences to investigate the relationship between HIF-1α and SIRT1 expression.

## Electronic supplementary material

Supplementary information
