# Supplementary material for: Hypoxia-inducible factor 1 alpha promotes cancer stem cells-like properties in human ovarian cancer cells by upregulating SIRT1 expression
Source: Sci Rep. 2017 Sep 6;7:10592. doi: 10.1038/s41598-017-09244-8 (PMC5587562; doi:10.1038/s41598-017-09244-8)
Supplement: Supplementary file 1 — Supplementary information [file 41598_2017_9244_MOESM1_ESM.pdf]

Research article

Hypoxia-inducible factor 1 alpha promotes cancer stem cells-like properties in human ovarian cancer cells by upregulating SIRT1 expression

Running title: Hypoxia-inducible factor 1 alpha promotes cancer stem cells-like properties

Authors: Jie Qin [#*1], Yan Liu [#2], Yongkui Lu [2], Meiling Liu [2], Manli Li [2], Juan Li [2], Lijuan Wu[2]

Authors' affiliations: [1]Obstetrics and gynecology, Center for reproductive medicine and genetics. The people's hospital of Guangxi Zhuang Autonomous Region.

[2]The Department of breast and bone tissue oncology, Affiliated Tumor Hospital of Guangxi Medical University, Nanning, People's Republic of China.

Corresponding author: Jie Qin , Obstetrics and gynecology, Center for reproductive medicine and genetics. The people's hospital of Guangxi Zhuang Autonomous Region. No.6 Taoyuan Road, Nanning, Guangxi province, 530021, China. Tel: 86-0771-2804224, Fax: 86-0771-2802018

[#] These authors contributed equally to this work.

Figure 1

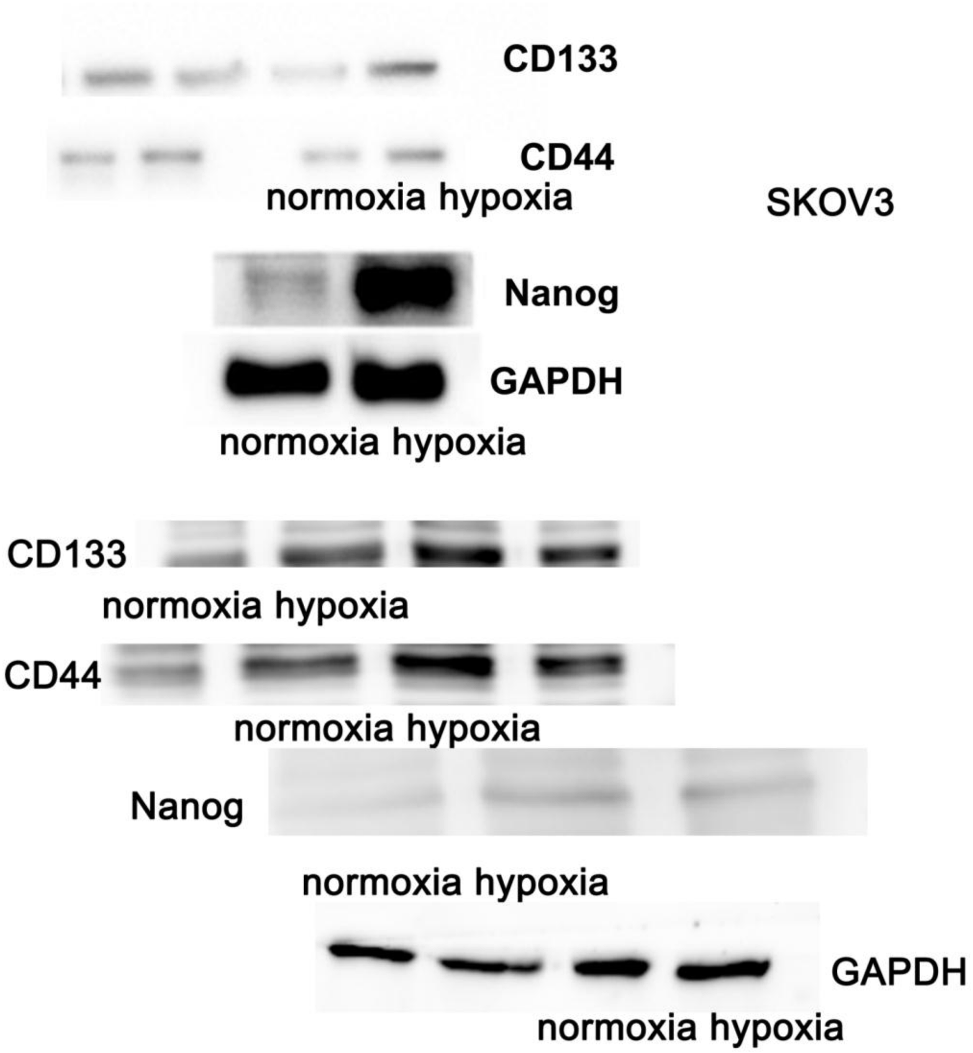
CD133
CD44
normoxia hypoxia
SKOV3
Nanog
GAPDH
normoxia hypoxia
CD133
normoxia hypoxia
CD44
normoxia hypoxia
Nanog
normoxia hypoxia
GAPDH
normoxia hypoxia

Figure 5

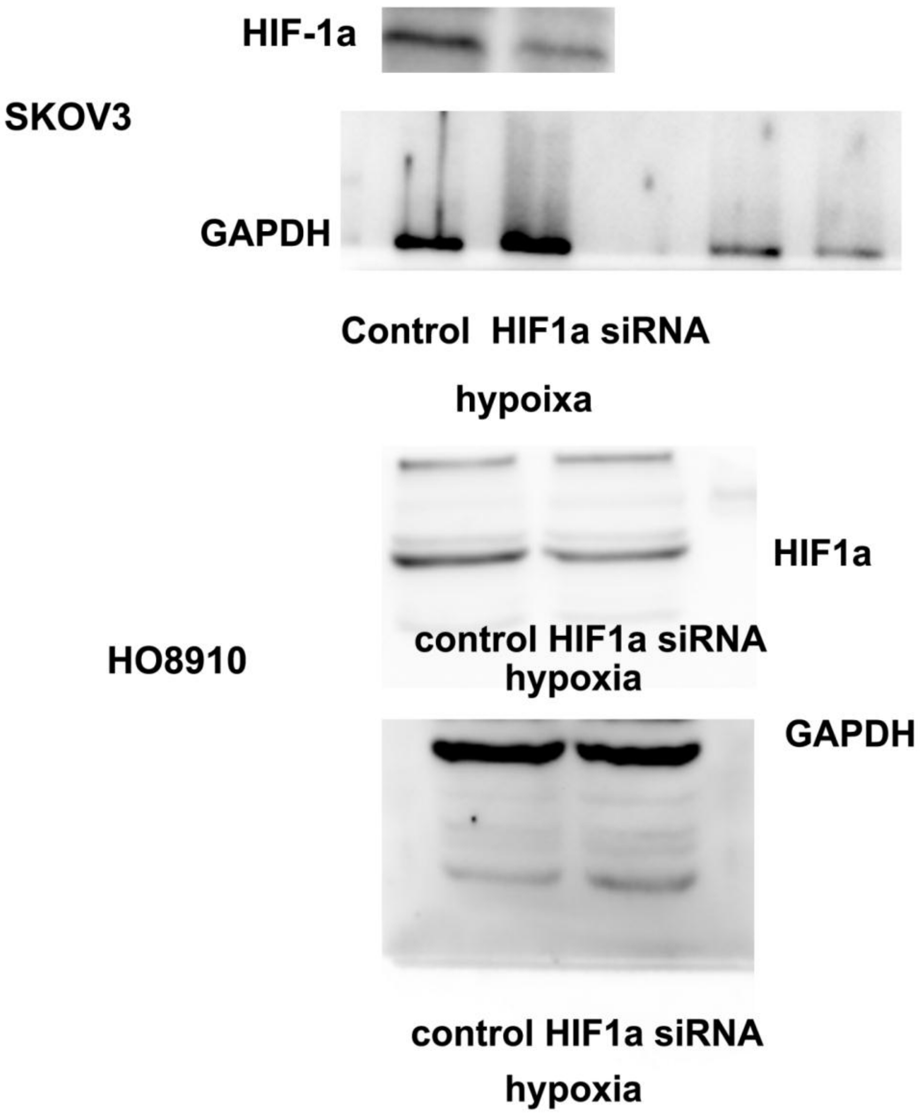
HIF-1a
SKOV3
GAPDH
Control HIF1a siRNA
hypoixa
HIF1a
HO8910
control HIF1a siRNA
hypoxia
GAPDH
control HIF1a siRNA
hypoxia

Figure 7

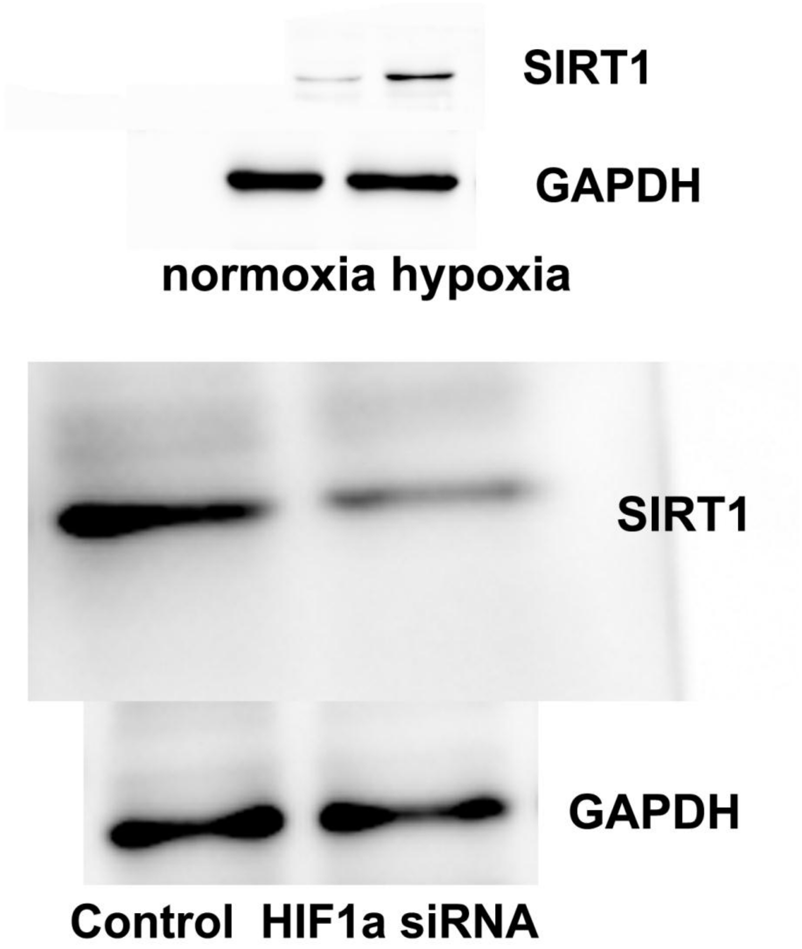
SIRT1
GAPDH
normoxia hypoxia
SIRT1
GAPDH
Control HIF1a siRNA

Figure 8

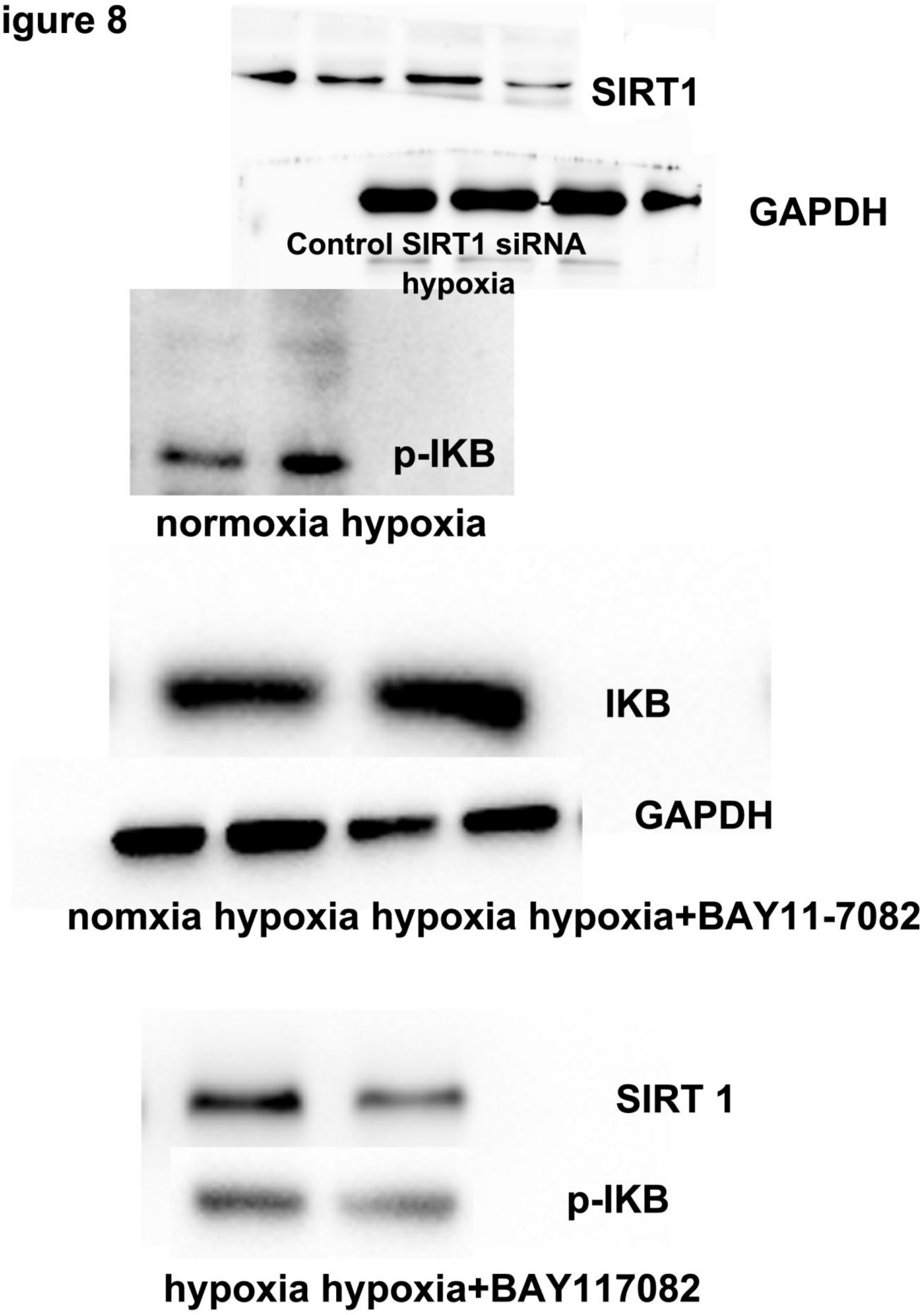

SIRT1
GAPDH
Control SIRT1 siRNA
hypoxia
p-IKB
normoxia hypoxia
IKB
GAPDH
nomxia hypoxia hypoxia hypoxia+BAY11-7082
SIRT 1
p-IKB
hypoxia hypoxia+BAY117082
